# Improving Equity of Access Through Electronic Consultation: A Case Study of an eConsult Service

**DOI:** 10.3389/fpubh.2019.00279

**Published:** 2019-10-04

**Authors:** Clare Liddy, Justin Joschko, Sheena Guglani, Amir Afkham, Erin Keely

**Affiliations:** ^1^C.T. Lamont Primary Health Care Research Centre, Bruyère Research Institute, Ottawa, ON, Canada; ^2^Department of Family Medicine, University of Ottawa, Ottawa, ON, Canada; ^3^Ontario eConsult Centre of Excellence, The Ottawa Hospital, Ottawa, ON, Canada; ^4^The Champlain Local Health Integration Network, Ottawa, ON, Canada; ^5^Department of Medicine, University of Ottawa, Ottawa, ON, Canada; ^6^Division of Endocrinology/Metabolism, The Ottawa Hospital, Ottawa, ON, Canada

**Keywords:** eConsult, access to care, equity, low resource, complex circumstances, health inequality

## Abstract

**Background:** Patients with complex circumstances pertaining to geography, socioeconomic status, or functional health often face inequities in accessing care. Electronic consultation (eConsult) is a secure online application that allows primary care providers (PCPs) and specialists to communicate regarding a patient's care. eConsult has demonstrated an ability to improve access to specialist care, and may be of particular use in cases of inequitable access.

**Methods:** We examined how eConsult is used to improve equity of access for patients in complex circumstances by conducting a multiple case study of eConsults from seven patient groups: addiction, frail elderly, homeless, long-term care, rural, special needs, and transgender. Cases from these groups were selected from all eConsult cases completed between January 1 and December 31, 2017 using a data collection strategy tailored to each group. An access framework by Levesque et al. was applied to the data to examine five dimensions of access, arranged in chronological order, that reflect the process of a patient seeking care: approachability, acceptability; availability, affordability, and appropriateness. Two reviewers analyzed the cases using an iterative approach, regularly presenting findings to the research team for discussion and interpretation.

**Results:** Eight hundred and twenty-five cases emerged across the seven target groups. The selected cases highlighted a number of key factors, including the value of the patient-PCP relationship, the importance of considering patient perspectives when providing care, and efforts to accommodate patients facing particular challenges to accessing care. Examples emerged among all five dimensions of the Levesque et al. access framework, with the final dimension, appropriateness, emerging across all cases.

**Conclusions:** By leveraging the eConsult platform, PCPs can help improve equitable access to specialist care. More research is needed to understand why patients with complex circumstances face a longer wait time compared to the general population, and the impact that eConsults can have in improving health outcomes and wait times for this population.

## Introduction

While Canada provides universal healthcare coverage for all citizens, many Canadians nevertheless face inequities in their interactions with the healthcare system (1, 2). Inequities in healthcare refer to differences in health status, outcomes, and levels of access between patients in different population groups (3). Inequity can manifest in populations of patients with complex circumstances caused by a range of economic, social, and demographic factors, including age, socioeconomic status, cultural or racial background, sexual orientation, and functional health (e.g., physical frailty, mental impairment) (4).

A recent report by the Pan-Canadian Health Inequalities Reporting Initiative explored the inequities faced by various patient groups, stratifying by socioeconomic status, place of residence (i.e., rural vs. urban), and population group (e.g., age, sexual orientation, functional health, cultural background). The report found that patients in each of these groups face numerous inequities in care, resulting in a higher incidence of chronic conditions (e.g., asthma, diabetes, arthritis), worse health behaviors (e.g., increased smoking and alcohol consumption), and lower overall life expectancy (4). Among the report's chief recommendations was an emphasis on ensuring “equitable access to opportunities for health, well-being, and their determinants” (4). Equitable access to care is a core component of overall equity, as it determines whether and how easily patients with various complex circumstances can seek the care necessary to improve health outcomes.

Electronic consultation (eConsult) is a means of secure online communication that lets primary care providers (PCPs) such as family physicians and nurse practitioners connect with specialists regarding a patient's care (5). Studies of eConsult services worldwide have demonstrated their ability to improve access to specialist advice through prompt response times and an ability to resolve many cases without the patient requiring a face-to-face specialist visit (5, 6). One such program is the Champlain BASE™ (building access to specialists through eConsultation) eConsult service, which was launched in 2010 in Eastern Ontario. The eConsult service has proven its ability to address the Quadruple Aim of healthcare outcomes by delivering better population health (e.g., improved access to specialist advice, reduced specialist visits), an improved patient experience of care (e.g., increased patient satisfaction), increased provider satisfaction (e.g., reports of educational value), and lower costs (e.g., lower per capita cost of eConsult vs. in-person specialist visit) (7).

Evidence has begun to emerge suggesting that eConsult and other telemedicine innovations can improve equity for populations with complex circumstances (8–11). A recent study conducted in Rochester, New York examined rates of access to care use between inner city and suburban children before and after the introduction of a telemedicine service. The study found that children in higher income suburban neighborhoods had 75% more clinic visits than children in lower-income inner city neighborhoods at baseline, whereas after the service was implemented, the gap shrunk to statistical insignificance, suggesting a redress in the inequity of access between the two communities (8). Likewise, studies of telemedicine services operated by Médecins Sans Frontières and the United States Military in low-income countries, including South Sudan, the Democratic Republic of the Congo, Ethiopia, Iraq, and Yemen, demonstrated improvements in access to and quality of care that can help to address the overall care inequities faced by patients in these countries (9–11).

There is reason to believe eConsult can have a similar impact on equity. By connecting PCPs and specialists electronically, eConsult provides an alternative vehicle to specialist advice for patients whose complex circumstances make attending a face-to-face specialist appointment difficult. An elderly patient in a nursing home may be too frail to travel to a specialist, or have to rely on family or friends to bring them to the appointment. A patient with complex co-morbidities or physical or mental impairments may require specialized transportation, such as an ambulance. Patients in rural regions may have to travel significant distances to attend specialist appointments, as many specialties operate exclusively in large urban centers. Furthermore, some patients may be more comfortable accessing care from their own primary care provider than from a specialist they have not previously met operating from an unfamiliar clinic. Our own preliminary findings support this view; early descriptive studies of eConsult's use among elderly patients, patients suffering from chronic pain, and those living with HIV suggest that eConsult could be closing a care gap and improving equity of access for these population (12–14). However, further study is needed to assess whether the benefit offered by eConsult extends to other groups of patients whose complex circumstances may cause them to face inequality of access.

Given eConsult's potential to reduce geographical and cultural barriers by connecting providers and alleviating patients' burden in navigating the healthcare system, we sought to gain a richer understanding of the service's impact on equity. In this study, we examined how eConsult is used to improve equity of access for patients in complex circumstances by conducting a multiple case study of eConsults for patients from seven potentially underserved groups.

## Methods

### Design

We examined eConsult cases sent on behalf of patients with complex circumstances using a multiple case study design (15). We selected this design for two main reasons. First, as outlined by Yin, case studies are ideally suited to pursue answer to “how” and “why” questions that cannot be adequately addressed through quantitative means (15). As such, this design allowed us to explore how eConsult may improve equity of access for populations with complex circumstances. Second, Yin's methodology emphasizes how case studies provide an effective approach when attempting to examine a natural phenomenon, as they offer a means to directly assess interactions and behaviors rather than gaining them secondhand through interviews or surveys. In the case of this study, said phenomenon is the eConsult itself. By reviewing the question posed by the PCP and the response provided by the specialist, we are effectively able to examine an interaction between healthcare providers without relying on the filter of secondhand accounts. The eConsult service keeps a complete log of their exchange, meaning we are privy to the entire dialogue with no omissions, allowing us great insight into these specific exchanges.

### Setting

The eConsult service is a web-based application hosted on a Microsoft SharePoint platform. PCPs log on using any device with internet access, enter their questions in a free-text field, attach any files they deem relevant (e.g., photos, test results) and select a specialty group. Specialists respond within 1 week with advice for the PCP, a recommendation to proceed with an in-person referral, or a request for more information. The PCP decides whether to respond with further questions or close the case.

We selected a preliminary dataset of all cases completed between January 1, 2017, and December 31, 2017. We chose this date range for two reasons. Firstly, it ensured that all cases included in our study were relatively recent. Second, it allowed us to observe a period during which case volume was sufficiently large—approximately 1,000 cases per month—and all participating PCPs had access to a roster of over 100 specialty groups.

### Participants

Within the 2017 dataset, eligible cases included those submitted for patients with a range of complex circumstances. Our team held a meeting on December 3, 2018 to discuss the parameters of the groups to be included in the study, drawing on their experience with eConsult and clinical care to target key populations whose complex circumstances can result in low equity of access. Participating team members included a practicing family physician (CL), an endocrinologist (EK), and an engagement and implementation lead (AA). We made an effort to select populations who had a high likelihood of access issues arising from a range of circumstances, including geography, medical complexity, and socioeconomic status. Seven groups were chosen by consensus:

1. Patients struggling with addiction.
2. Elderly patients with frailty.
3. Patients who were homeless.
4. Patients living in long-term care (LTC) facilities.
5. Patients living in rural or remote locations.
6. Patients with developmental disabilities, defined in this study as “special needs”.
7. Transgender patients.

### Inclusion Criteria

To be included in the study, the cases must have been completed during the 1-year data collection period (January 1, 2017 to December 31, 2017) and involve a patient fitting in one of the seven complex circumstance groups mentioned above.

### Theoretical Framework

**Figure 1:**
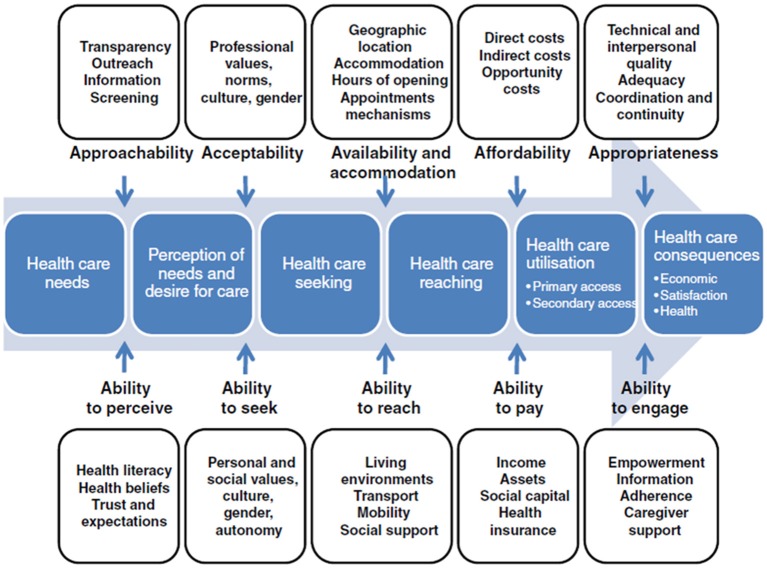
Model of the five dimensions of access defined by Levesque et al. (16). Figure has been reproduced from Levesque et al. (16) under the terms of the Creative Commons Attribution License (http://creativecommons.org/licenses/by/2.0) with permission from the original publisher, BioMed Central Ltd.

We approached this study through the lens of an access framework created by Levesque et al. which defines access as “the opportunity to reach and obtain appropriate health care services in situations of perceived need for care” (16). According to Levesque et al. access is not measured by a single point of contact, but describes a range of interactions between patients, caregivers, providers, and the healthcare system. To reflect this complexity, Levesque et al. posit five dimensions of access, arranged in chronological order, that reflect the process of a patient seeking care: approachability, acceptability; availability, affordability, and appropriateness. These “supply side” dimensions reflect the healthcare system's perspective, and each is paired with a “demand side” dimension articulating the patient's ability to access care: ability to perceive, ability to seek, ability to reach, ability to pay, and ability to engage (Figure 1).

### Data Collection and Analysis

We developed a data collection strategy for each of the seven target groups. For groups with specialties dedicated to their care (addiction, transgender), we selected all cases submitted to these specialties during the study period. For the LTC group, whose cases came from an identifiable subset of providers (i.e., PCPs working in LTC homes), we selected all cases submitted from these providers during the study period. For the remaining groups (frail elderly, homeless, rural/remote, special needs), we compiled a list of keywords pertaining to each group and used them to conduct a search of case logs for all cases completed during the study period. Other fields were used where appropriate to exclude irrelevant cases from the dataset (e.g., for frail elderly, only cases submitted for patients aged 75+ were included in the keyword search). A complete list of the search criteria and keywords used is available in Appendix A in Supplementary Material.

A team member with experience conducting qualitative analyses (SG) performed the keyword searches and extracted the data for all seven groups. A second team member who also had previous experience conducting qualitative analyses (JJ) reviewed all cases, excluding those that did not involve patients from the target group or that lacked sufficient detail to inform a case study. The reviewer assessed all cases included at this stage a second time, from which he selected a shortlist of 2–3 cases per group. Cases at this stage were deidentified, with all reference to patient and provider names removed to prevent possible identification. Two senior researchers with a combination of research and clinical experience (CL and EK) reviewed the shortlist and made a final selection for each group on the basis of clinical relevance. Considerations used to select the final dataset included: (1) the richness of detail in the case log, and (2) stratification to ensure diversity of (a) specialty group, (b) patient demographics (e.g., age, gender, background), and (c) type of advice (e.g., diagnosis, suggestion for medication, treatment strategy).

SG and JJ reviewed all included cases separately. They applied the Levesque et al. framework to each case, identifying which of the dimensions emerged from the case content. The reviewers met to compare their findings and resolved any discrepancies by consensus. Their findings were presented to the research team for discussion and interpretation. Cases at this stage were further deidentified to remove any reference to patient ages, locations, or events deemed by the research team to be too specific and potentially identifying.

### Research Ethics Approval

The Research Ethics Board of the Ottawa Health Science Network provided ethics approval for analysis of the full eConsult dataset, which includes the analysis performed in this study (Protocol #: 2009848-01H). This study functions as a subset of our larger project, drawing on data collected automatically through the eConsult service's communication logs. Patient consent could not be collected, since the research team has no direct contact with any of the patients treated using the eConsult service, and cannot obtain their contact information for privacy reasons. All cases were rendered fully anonymous through the removal of any identifying information in order to safeguard patient privacy.

## Results

**Figure 2:**
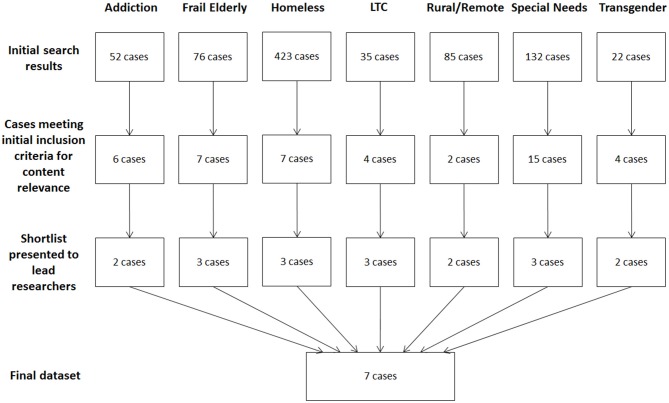
Flow chart of data collection strategy.

A total of 825 cases across the seven target groups emerged from the initial search (Figure 2). A complete review of the results yielded between two and 15 cases per group that met preliminary approval for relevance and richness of content. Instances where multiple eConsults referred to the same patient issue were grouped as a single case. A second review of these cases narrowed the potential targets to between two and three cases per group, from which a single case from each group was selected. The total sample was thus eight eConsults pertaining to seven cases, as the final case chosen for one group (LTC) spanned two eConsult exchanges.

### Addiction Case

The patient was a woman with a longstanding history of chronic back pain, which multiple surgeries had failed to alleviate. She has used opioids to manage her pain for many years. Previous efforts to eliminate usage through methadone failed, and she recently reacted negatively to the PCP's efforts to reduce dosage. Furthermore, the PCP noted that “she has repeatedly violated her narcotic agreement contract by requesting early refills for questionable reasons, failing to submit regular urine drug screen samples, occasionally testing positive for [illegal substances].” Consequently, the PCP is no longer comfortable prescribing opioids for her. The PCP referred the patient to an addiction treatment center, but noted “she did attend, but was told that they don't prescribe methadone for chronic pain patients, so the problem appears to be back in my lap.” The PCP remained against prescribing further opioids, and asked the specialist for advice.

The specialist acknowledged that the PCP was in a difficult position, as “the patient is pre-contemplative for change: you feel there needs to be a change but she is very attached to the status quo.” Based on the PCP's description, the specialist stated that the patient likely has Opioid Use Disorder (OUD) and supported the PCP's decision not to prescribe opioids: “You are entitled to hold to your boundaries of what your judgement tells you is safe and effective. Continued [opioid use] is neither safe nor effective.” Instead, the specialist recommended “pharmacologic OUD treatment [methadone or Suboxone] and behavioral boundaries.” The specialist underscored the importance of pharmaceutical intervention, noting “it's like she went off her diabetes medication, and now there is a crisis because her blood sugars are high.” The specialist acknowledged that such an encounter will likely be challenging, and that there were limits to the PCP's ability to control the patient's behavior:

*Fundamentally the ball is in the patient's court. You cannot continue the status quo, because in your judgement it is not safe or effective. She is free to disagree, but her disagreement need not change your judgment. She can go back to [the addiction treatment centre] again for methadone or Suboxone once she has accepted that addiction is dominant over her pain currently. Any other clinic would give her the same message*.

The specialist concluded by providing contact information for their own clinic, noting that while their practice was currently full, space may open up in the near future. Additionally, the specialist suggested that “regardless if an opioid is prescribed or not, she should access a free naloxone kit from her pharmacy in case of overdose.”

### Frail Elderly Case

The patient, an elderly man, had experienced multiple falls: “[patient] was admitted to [general hospital] for a few weeks with no cause found. He was there rehab'ed with [geriatric hospital] for another few weeks before being sent back to his RH [retirement home] with still a high fall risk.” The patient had applied for 24-h long-term care, but the application would likely take several months to process, and in the meantime the patient remained at home. Previous efforts to diagnose the cause of falls had been unsuccessful, including a referral to a neurologist that the family stopped seeing, as they “apparently found the [physician's] manner very off-putting.” The PCP expressed concern, as “at this point, every time he falls he is at high risk of hurting himself,” and asked “if there is an outreach geriatric consult I could refer to, or if [specialized care team for geriatric patients] might be an option?”

The specialist recommended sending the patient to a local geriatric day hospital for outpatient treatment if appropriate. The PCP expressed reluctance to refer to an outpatient clinic: “I actually am not sure he's even in good enough shape for the day hospital.” The specialist recommended calling the discharging doctor at the hospital to discuss options:

*If he is too weak to attend any ambulatory care services and cannot leave home then I would ask the discharging MD if they can help arrange a re-admission to [geriatric hospital] as this would represent a failed discharge so there is some shared responsibility. The discharging MD should have more leverage through internal conversations within [the hospital]*.

### Homeless Case

In this case, the PCP reached out for advice regarding a patient with alcohol use disorder alongside other issues related to anxiety and depression. The patient “was being seen by psychiatry back in [date] and again in [date several years later] while she was a [college] student” and has tried a number of antidepressants. Despite these efforts, the patient continued to struggle:

*In the 1 year that she has been my patient she has been to ER over 25 times either in withdrawal or because of alcohol related issues. […] She has become homeless and has repeatedly tried to get her life back on track but relapses. She has a counsellor, has been in rehab multiple times and I have encouraged her to go to the [hospital] on self-referral basis for the addictions and mood disorder clinic but I don't believe she has*.

The PCP expressed concern for the patient, as “I see her getting more and more hopeless and feel at a loss for what to do.” The PCP noted that they had not yet tried “naltrexone or other pharmaceutical agents for alcohol abuse,” and sought advice on this and any other recommendations.

The specialist encouraged the PCP to try alcohol use inhibitors and provided several suggestions. For each, the specialist offered detailed guidance on dosage, possible side effects, and requirements for abstinence from alcohol and opioids prior to use. Additionally, the specialist offered detailed guidance on how to access the medication through the Ontario Drug Benefit's Extended Assistance Program, which allows low-income patients to receive medication without cost to them: “There is a sample EAP [Extended Assistance Program] form on the META:PHI site: you can fill in the patient's name, print, sign, and fax it. It sometimes takes a few weeks but they always have said yes.”

The specialist also provided contact information for a local managed alcohol program that could be useful for the patient: “If your patient has been homeless, she is eligible for this program. It provides housing and a scheduled supply of alcohol, so the patient doesn't have to binge. She can still access all other treatments within the [program].”

### Long-Term Care Case

The patient was a woman with schizoaffective disorder who experienced regular visual hallucinations. She took a number of antidepressants to manage her condition, and had previously consulted with a psychiatrist and neurologist, though she refused to return to her previous psychiatrist or access psychiatric services at the emergency department. The PCP sent eConsults simultaneously to psychiatry and neurology, posing the following questions: “(1) how can we safely optimize medical/ pharmacological management of patient's hallucinations, [and] (2) can you provide us with contacts/resources for continued long-term community follow up?” In addition, for the neurology eConsult only, the PCP mentioned persistent headaches, described as “10/10 during the day and worsens overnight,” and sought advice on their treatment.

The psychiatrist responded with a detailed program of medications for the PCP to prescribe, including antipsychotics as well as dietary supplements such as B12 and folate. For the medications, the psychiatrist outlined dosages, timelines, and side effects to watch for, noting:

*With all of these changes, it will be important to have a good understanding of what her baseline is in terms of positive, negative, and cognitive symptoms. Hopefully you have a sense of this or can get it from records. This will help to set realistic goals in terms of what she looks like when she is well (e.g., if she still has some baseline hallucinations but is much less distressed and more organized)*.

The psychiatrist also addressed the PCP's request for resources, noting “The [local hospital] is really the only place that provides that kind of chronic care. If she has had multiple hospitalizations she could be eligible for an ACTT team however the wait list is ~1 year or more. If she is French speaking, the [local francophone hospital] does provide some outpatient consultation and treatment.”

The neurologist responded by pondering the accuracy of the patient's self-assessment: “I question someone walking into the office with 10/10 headache and being able to function. The visual hallucinations are a concern, as are many of the other symptoms that you described that most readily fit with a psychiatric, not neurological, condition.” The neurologist requested the patient's name in order to look up her records at the hospital.

The PCP supplied the name, and the neurologist replied with details of the patient's referral history. The neurologist noted that the patient had undergone numerous CT scans for damage resulting from [a traumatic neurological event] as a teenager, and provided the name of the neurologist who had seen her in the past, suggesting “perhaps having her see him again, since he knows her and the problem, he might indicate whether they are the same rather starting all over again.”

### Rural/Remote Case

The patient in this case was a woman who regularly worked in Northern Canada. During her latest trip, she had borrowed sunscreen from a co-worker and developed hives. Antihistamines helped alleviate the outbreak. The PCP provided pictures and asked for advice on diagnosis and treatment, noting the patient “will continue to be in very remote areas where any medications needed will need to be prescribed ahead of time.”

The specialist responded with an interpretation of the pictures, which showed “urticarial papules on the cheeks and dorsum of the fingers. The differential includes polymorphous light eruption and contact urticarial.” To avoid future outbreaks, the specialist proposed the patient “do a challenge test with the same sunscreen by applying some to a small area of her forearm and check for any rash.” The specialist suggested prescribing topical steroids for her face and hands to use alongside antihistamines, and advised having the patient “identify a good broad-spectrum sunscreen that she tolerates: [Particular brand] would be a good one to try.”

### Special Needs Case

In this case, the PCP reached out in regards to a young patient who had recently arrived as a refugee with his family. The PCP began the consult by noting “I am not sure if there are any reasons to be concerned about [the patient]. However, he is new to Canada and I feel like he deserves to be assessed by a Pediatrician once in case there are issues which need a higher level of support.”

A main point of concern was that the patient's “head circumference is only [number well below average], and he may have a bilateral ptosis.” The patient's older brother had a similar head circumference alongside developmental delays, suggesting a possible genetic component. The patient's mother denied noticing any developmental issues in the patient, though his minimal education made this hard to determine: “[patient] does not read or write. He only went to school [for a short period], and then dropped out because he didn't like it. He would go to his father's work, but he never really did anything there. He does not really have any hobbies.”

The PCP noted that the patient had been referred to genetics and dual diagnosis clinics at the local hospital, with the latter referral prompted because “apparently the brother was diagnosed with [psychiatric condition] after [a traumatic incident], and has not been normal since then.” The PCP concluded that “in summary [patient], seems to be a child who grew up without access to medical care, in a dangerous environment. He and his brother both have very small heads, and one wonders if there may be a Genetic condition at play.”

The specialist agreed with the decisions to refer, though cautioned the PCP regarding wait times in some cases. For the dual diagnosis referral, the specialist was “not sure if they will see [the patient] if he doesn't have any symptomatology himself. They have a phone consult service with a shorter wait time, might be worth getting a phone consult.” In addition to the existing referrals and treatment plan, the specialist noted: “It sounds like this fellow has a nice PSR [Private Sponsorship of Refugees] group, would encourage them to get him involved in some age appropriate sports program at local community center which may have a preventative effect in regards to mental health.”

### Transgender Case

The patient was a Trans woman who had recently arrived in Canada as a refugee. She had started androgen blockers and estrogen ~3 months prior, though she no longer had them at the time of the appointment and did not recall which type of androgen blocker she used. The PCP had detailed discussions with the patient regarding goals, noting “I have gone through the Trans Health Guidelines from Sherbourne and the Trans Care Guidelines with her line by line, and have completed a Readiness Assessment with her. She meets the criteria for Gender Dysphoria.” Having completed an assessment, the PCP intended to resume androgen blockers and asked “if you would suggest Spironolactone, or Cyproterone? At which dose?”

The specialist recommended spironolactone as a first-line androgen blocker, as “it is covered under ODB [Ontario Drug Benefit] and generally well-tolerated,” and provided details on dosage and monitoring. The specialist followed this advice with instructions on when and how to add estrogen to the schedule, as well as guidance on securing it for the patient without direct cost to her: “Apply for the EAP [Exceptional Access Program] for [estrogen] ahead of time. There is a sample form at the back of the SHC guidelines. The approval is generally pretty fast.”

If the spironolactone proved ineffective, the specialist suggested trying cyproterone as a second-line drug, and provided dosage instructions in similar detail. While expressing a preference for the former drug, the specialist qualified the recommendation with advice on how to customize based on patient risk factors: “Historically, we have used spironolactone as first line androgen blocker, but some providers now are feeling more comfortable using cyproterone before spiro. It's up to you. Just consider if the patient is more likely to be at risk of renal (avoid spiro) vs. liver side effects (avoid cyproterone).”

Lastly, the specialist concluded with “one quick note to mention before starting, also counsel on contraception—i.e., even on hormones, sperm production can occur, so she could still get someone pregnant theoretically. I always counsel on contraception.”

### The Levesque et al. Framework

#### Approachability

Approachability pertains to the patient's “ability to perceive,” meaning their awareness that a provider or service exists for them to access in the first place, as well as their level of health literacy, health beliefs, and trust in and expectations of the healthcare system. While the nature of the data made it difficult to observe whether eConsult helped inform patients about services with which they were previously unaware, references to other aspects of this dimension, namely patients' health literacy and expectations, emerged from several PCP questions we examined. Examples include the LTC case, when the PCP notes that the patient has indicated that she refused to return to her previous psychiatrist or access psychiatric services at the emergency department, and the transgender case, where the PCP indicates that the patient has expressed her desire to go back on her medication and has clearly defined her health goals.

#### Acceptability

Acceptability, or the “ability to seek,” means care that does not conflict with patients' personal, social, or cultural values, and that they can access without feeling unsafe or uncomfortable. It also includes issues that influence a patient's autonomy and capacity to seek care. We perceived this dimension in several cases where the PCP emphasized their relationship with the patient and took pains to ensure their concerns were answered and preferences respected, as well as where services may have already been accessed and were deemed unacceptable for the patient. This was particularly evident in the transgender case, where the PCP informed the specialist of the patient's preferences for transition and desire to eventually have children. In the LTC eConsult, the PCP hinted at the fact that the patient might have lost autonomy as a result of her hallucinations and cognitive impairments.

#### Availability

Availability, or “ability to reach,” involves a patient's capability to attend appointments, receive treatment, or otherwise access care, a process that can be negatively affected by complex circumstances. In the case studies, availability typically emerged through specialist's consideration of the patient's unique circumstances, and pursuing avenues of treatment that, where possible, limited their burden of accessing care. For instance, in the frail elderly case, the PCP carefully considered of the patient's ability to travel when discussing next steps, and offered the PCP guidance on the most appropriate avenues of referral for the patient. Furthermore, the Addiction case provides an interesting variation of this dimension, as the specialist suggests avenues of treatment (i.e., ceasing prescription of opioids, providing pharmaceutical support and behavior change) that likely go against the patient's desires, but are nevertheless in the patient's best interests.

#### Affordability

Affordability, or ability to pay, considers the financial issues that may affect a patient's ability to access care. These include the direct cost of services themselves, as well as secondary costs involved in reaching the appointment (e.g., travel, childcare), and opportunity costs associated with seeking care (e.g., missing work). This dimension was most commonly reflected in the specialist's recommendations for medication, which in three different cases (homeless, LTC, and transgender) included specific instruction on how to access the prescription through the Ontario Drug Benefits program without incurring cost to the patient.

#### Appropriateness

Appropriateness, or “ability to engage,” considers the alignment of services with the patient's needs. It includes such aspects as interpersonal relationships, timelines, and coordination of care. This dimension emerged as a reflection of the eConsult service across cases, as it addressed eConsult's ability to provide appropriate, prompt, well-coordinated care for patients.

## Discussion

The seven cases highlighted in our study provide insightful examples of eConsult's ability to address inequities of access among patients with complex circumstances. The Levesque et al. framework was helpful in guiding our review of the selected cases and the impact on equity of care they presented.

The cases included in this study were chosen to reflect populations that are particularly susceptible to inequities of access to health care services. Barriers to access are of particular concern to patients with complex circumstances pertaining to issues such as geography (e.g., patients from rural, remote regions), age (e.g., elderly patients), socioeconomic status (e.g., homeless patients), and comorbidity (e.g., patients in long-term care homes). Studies have shown that members of these populations face a number of significant barriers extending from their particular circumstances, which result in inequity of care. For instance, studies of access to care in rural regions have highlighted a number of barriers that are unique to, or more prominent among, communities outside of large urban areas, including fewer trained physicians, a scarcity of available services, poor public transit, less access to broadband internet, and a culture of stoicism (17, 18). Likewise, patients of low socioeconomic status face their own barriers to care access, including a lack of health insurance or benefits, a complicated registration process (particularly among patients with no fixed address), negative attitudes among providers, and the necessity of focusing on other needs at the expense of managing their health, such as seeking food and shelter (19–21). Other populations with complex circumstances report their own barriers to access, such as the stigma often faced by transgender patients (22–24) or those suffering from addiction (25, 26), or the anxiety and communication issues experienced by patients with developmental disorders (27, 28). In the cases highlighted by our study, eConsult proved an effective way to mitigate the inequities commonly experienced by patients in these populations.

The Levesque et al. framework offered an insightful way to examine the cases included in this study. Notably, eConsult's function as a communication tool for providers meant that the appropriateness dimension was present in all cases, since it pertained to the coordination of care that eConsult directly facilitates. This finding underscores the importance of ensuring that patients have a dedicated PCP, as patients who lack this essential touchstone are limited in their ability to access not just family medicine, but services in the broader healthcare community as well. Despite the importance of primary health care, 15.8% of Canadians report that they do not have a regular health care provider (29). Furthermore, studies have demonstrated inequities in access to family medicine for several of the groups highlighted in our study. For instance, a study focusing on access to family medicine among transgender patients found lower rates of family physician access among indigenous and homeless individuals (30). Another study assessing the experience of accessing family medicine in Canada among refugees—a group not deliberately included in our study, but which featured in two of the chosen cases (i.e., special needs and transgender)—highlighted several challenges, including a sense of futility and a lack of autonomy in the healthcare process (31). Likewise, an examination of perspectives from rural patients revealed financial challenges, difficulties in maintaining a relationship with a provider, and frustration with inefficiencies exacerbated by distance (32).

In recognition of this issue, governments in many jurisdictions have focused on improving access to family medicine by embracing a model of care called the Patient Centered Medical Home (PCMH), which empowers PCPs to remain central in a patient's care (33–36). In Canada, a similar model called the Patient's Medical Home (PMH) has been endorsed by the College of Family Physicians, and is reflected in the proliferation of clinics offering team-based models of care, such as Family Health Teams in Ontario and *Groupes de Médecine de Famille* in Quebec (35). By linking providers through a prompt and secure medium of communication, eConsult is a natural extension of the PCMH/PMH model, as well as the broader Patient Centered Medical Neighborhood, which specifically emphasizes the role of specialty medicine (37, 38).

Our study has several limitations. First, by using a case study model, we gained a detailed look at specific instances of care at the expense of generalizability. While the picture these cases paint is an encouraging one, we can make no broader statement on eConsult's impact on care equity. Further study examining eConsult's impact on measures of inequity (e.g., acute care visits, health outcomes) between population groups is warranted. Second, the nature of the data we used meant that our cases ended at the moment the PCP chose to close the eConsult. As such, we could not determine whether and to what extent PCPs followed specialists' advice, nor examine any follow-up conversations between the PCP and the patient. Though a recent study by our team found that PCPs adhered to specialist advice in 82% of cases (39), the nature of the data nevertheless limits our ability to interpret outcomes. Additional case studies that include patient and PCP follow-up through interviews and access to health administrative data would be illuminating. Third, when applying the Levesque et al. framework to the data, we were able only to assess its “supply side” dimensions reflecting the health system perspective. The Framework also posits a set of “demand side” dimensions, which mirror the supply side dimensions but address the patient's perspective (see Figure 1). These dimensions could only be explored through direct interaction with patients, which makes an intriguing premise for further study. Fourth, our data selection process was not random, and as such is vulnerable to selection bias. We sought to mitigate this issue by relying on a data extraction strategy using keywords and emphasizing maximum variation between cases. Lastly, the study involved cases from an eConsult service operating in a single province, with the majority of cases drawn from a single health region where the service is most prevalent, limiting the generalizability of its findings.

## Conclusion

Access to specialist care and equity of care are ongoing challenges for patients in the healthcare system, particularly for patients in complex circumstances pertaining to geography, socioeconomic status, demographics, and functional health. By leveraging the eConsult platform, PCPs can help improve equitable access to specialist care, and specialists are able to provide valuable advice on biomedical complexities, relationship building between patients and providers. However, access to a PCP is vital for patients to benefit from the improved access provided by the eConsult service. More research is needed to understand why patients with various complex circumstances face a longer wait time compared to the general population, and the impact that eConsults can have in improving health outcomes and wait times for these groups.

## Data Availability Statement

The deidentified data supporting the conclusions of this manuscript will be made available by the authors, without undue reservation, to any qualified researcher.

## Ethics Statement

The Research Ethics Board of the Ottawa Health Science Network provided ethics approval for this study (2009848-01H).

## Author Contributions

CL and EK conceived of the study, contributed to study design, and data analysis. JJ contributed to study design and data analysis. SG collected and analyzed the data. AA helped develop the data collection strategy and contributed to data analysis. All authors helped write the manuscript and approved a final copy of the manuscript.

### Conflict of Interest

CL and EK are Co-Executive Directors of the Ontario eConsult Centre of Excellence, funded by the Ontario Ministry of Health and Long-term Care. They co-founded the Champlain BASE™ eConsult service but do not receive any reimbursement or retain any proprietary rights. EK answers eConsults through the service, <1 per month. The remaining authors declare that the research was conducted in the absence of any commercial or financial relationships that could be construed as a potential conflict of interest.
