# Supplementary material for: Improving Equity of Access Through Electronic Consultation: A Case Study of an eConsult Service
Source: Front Public Health. 2019 Oct 4;7:279. doi: 10.3389/fpubh.2019.00279 (PMC6787760; doi:10.3389/fpubh.2019.00279)
Supplement: Supplementary file 1 [file Table_1.DOCX]

**Appendix A.** Keywords used in eConsult database search to identify cases from seven target groups.

| **Category** | **Subset of 2017 Cases to Search** | **Search Terms** |
| --- | --- | --- |
| Rural/remote | Cases from Nunavut, cases submitted from rural postal codes | Rural; remote; long distance; travel |
| Homeless | All cases | Homeless; transient; no fixed address; shelter; mission |
| Frail elderly | Patients age 75+ | Frail; immobile; mobility |
| Special needs | All cases | Special needs; autism; autistic; Down Syndrome; developmental delay; trisomy; developmental disability |
| Addiction | Cases sent to addiction specialty | [include all cases] |
| Transgender | Patients referred to transgender specialty | [include all cases] |
| Long-term care | Cases from PCPs practicing at LTC homes | [include all cases] |

PCP = primary care provider; LTC = long-term care
